# Staphylococcal scalded skin syndrome in a 4-year-old child: a case report

**DOI:** 10.1186/s13256-017-1533-7

**Published:** 2018-01-29

**Authors:** P. J. Haasnoot, A. De Vries

**Affiliations:** Department of Surgery, Burns unit, Rode Kruis Hospital, Beverwijk, Vondellaan 13, 1942LE Beverwijk, The Netherlands

**Keywords:** Staphylococcal scalded skin syndrome, SSSS, Burn center

## Abstract

**Background:**

Staphylococcal scalded skin syndrome is an exfoliating skin disease which primarily affects children. Differential diagnosis includes toxic epidermal necrolysis, staphylococcal scalded skin syndrome, epidermolysis bullosa, and Stevens–Johnson syndrome. Staphylococcal scalded skin syndrome primarily affects children and can cause serious morbidity.

**Case presentation:**

In this case report we highlight the case of a 4-year-old Caucasian boy. Diagnostic and therapeutic challenges are discussed. Differential diagnoses are considered and therapy is described and discussed. The latest treatment options are used and described. Successful results are achieved in this case due to timely and correct management.

**Conclusions:**

Some therapeutic options are widely used without thorough research bases. This case report highlights staphylococcal scalded skin syndrome and its treatment, and future challenges. Further research is warranted and this case report aims to further research in exfoliating skin disorders.

## Background

Multiple skin conditions present with blistering, such as toxic epidermal necrolysis (TEN), staphylococcal scalded skin syndrome (SSSS), epidermolysis bullosa (EB), Stevens–Johnson syndrome (SJS), and pemphigus. Due to different etiology and treatment, thorough history taking and examination is paramount. Furthermore, morbidity and mortality vary greatly. Therefore, a timely accurate diagnosis is important.

**Fig. 1:**
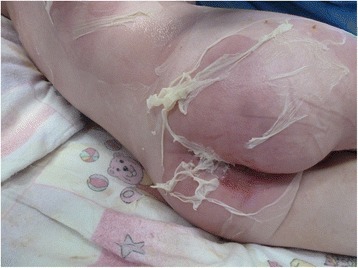
Superficial exfoliation of the lower back and gluteal region on day 3

**Fig. 2:**
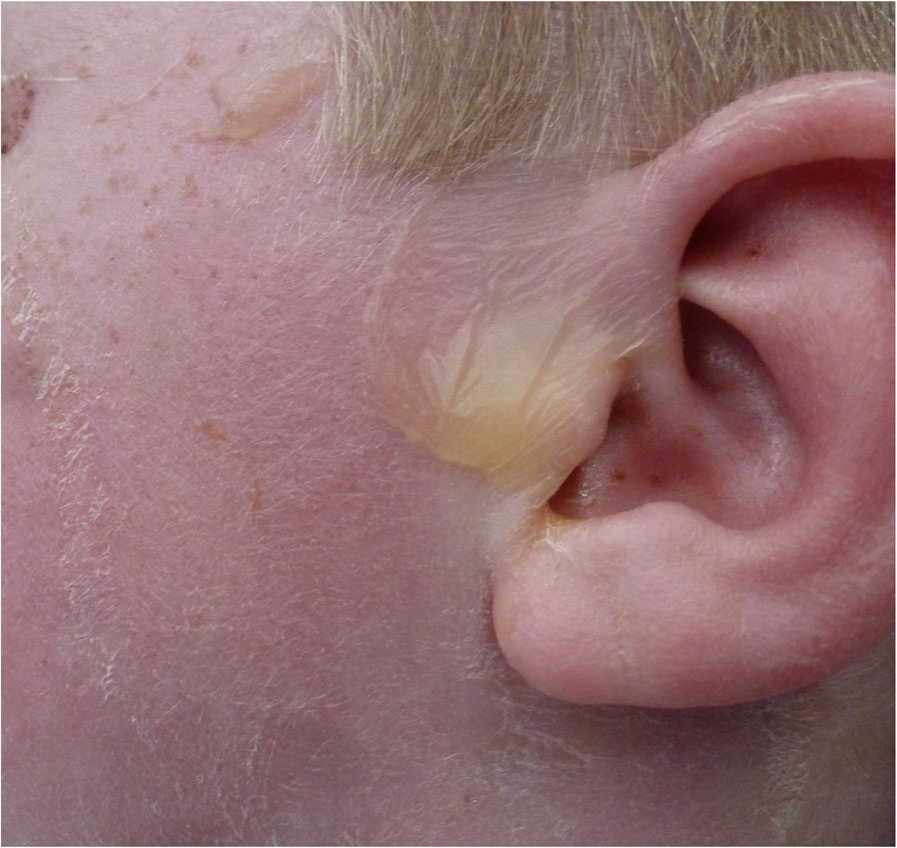
Superficial exfoliation of the face on day 3

**Fig. 3:**
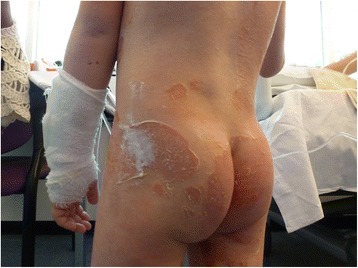
Healed exfoliation of the lower back and gluteal region on day 7

**Fig. 4:**
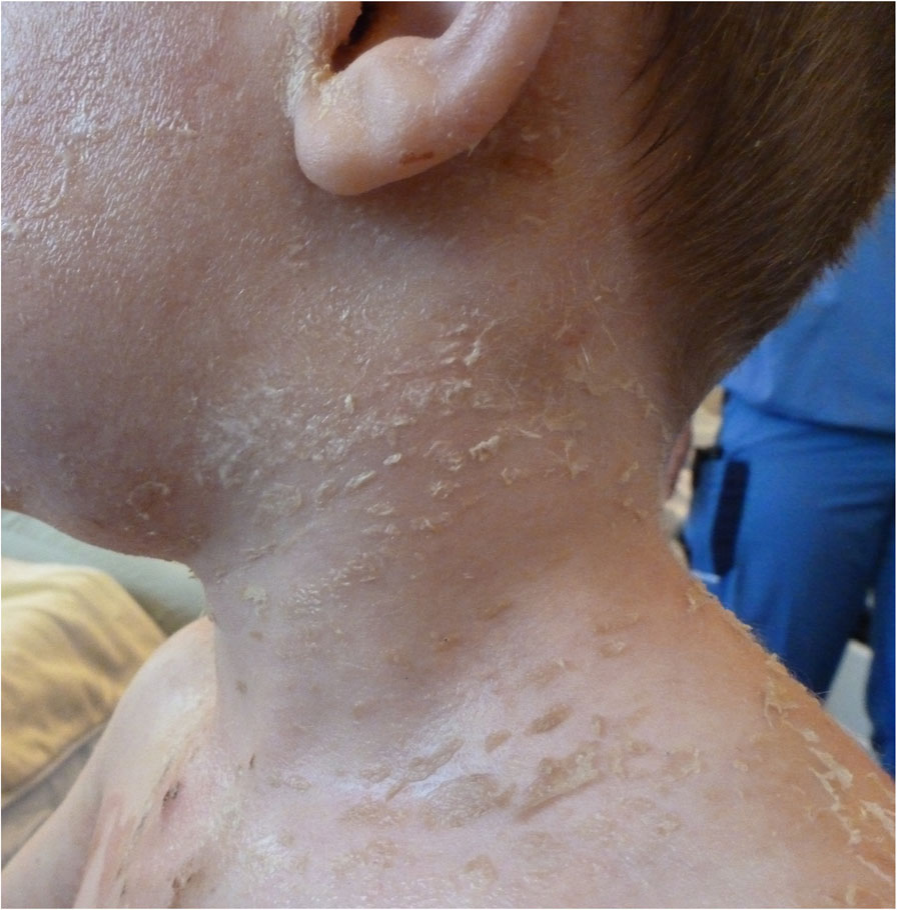
Healed exfoliation of the face on day 7

## Case presentation

### Days 1–4

We present the case of a 4-year-old Caucasian boy with a blistering skin syndrome. He had no relevant medical history and no use of medication prior to this event. No allergies were known and he had been vaccinated, according the Dutch vaccination program.

He presented to an emergency room with a history of loss of appetite, constipation, and agitation of 1-week duration. Furthermore, he experienced pain at his buttocks, lower back, thorax, and face. In addition, skin lesions started in the peribuccal area and appeared after rubbing of the skin (Nikolsky’s sign). At physical examination, erythema and exfoliation were present. He was apyretic. Mucous membranes were not affected. Exfoliation affected 10% of his total body surface area (TBSA). Laboratory tests revealed no signs of infections: leukocyte count (L) of 7.4 × 10^9^ and C-reactive protein (CRP) was 3 mg/l. Further investigation was performed, including skin cultures and biopsies.

Because of superficial scalding, Nikolsky’s sign, and no involvement of mucous membranes, SSSS was considered a working diagnosis and antibiotic treatment was initiated with intravenously administered flucloxacillin and clindamycin. His pain was managed with acetaminophen and morphine intravenously administered. Fluids management was monitored.

Other diagnoses such as TEN and impetigo vulgaris were considered. Since our patient’s mucous membranes were still not affected, SSSS remained most likely. He remained stable and pain was manageable. Figures 1 and 2 indicate exfoliation on day 3.

### Days 4–10

He was transferred to our Burns Unit in Red Cross Hospital in Beverwijk, the Netherlands. Most of the blisters had since resolved. The affected TBSA decreased to 5%. Antibiotic treatment was continued intravenously, and local analgesic therapy was added with Cavilon® (dimethicone) barrier cream. Clindamycin was continued to counter toxin secretion [1]. Feeding was managed via nasogastric tube. Our patient reported pain around his hands and feet. His leukocyte count remained normal: 7.3 × 10^9^/L. His CRP was slightly elevated: 23 mg/l.

During admission, his pain slowly subsided and exfoliation healed after 3 days without scarring. Exfoliation of his hands and feet developed some days after his report of pain but also healed within 3 days. After 5 days the antibiotics were administered orally, to a total of 7 days. Corneal exfoliation at the epidermis indicated superficial blistering. On day 10 he was discharged without complaints. Figures 3 and 4 show advanced healing on day 7.

#### Pathologic results

Extensive subcorneal exfoliation without inflammatory reaction or inflammatory cells, no keratinocytes, and no segmented granulocytes. Findings indicate SSSS.

#### Cultures

Common skin bacteria.

## Discussion

In this case, a prompt diagnosis of SSSS and treatment resulted in rapid healing and timely discharge. No complications or later complaints were reported.

SSSS is a rare skin condition which primarily affects children. It was first described by a German physician, von Rittershain, in 1878 [2], the staphylococcal infection causes superficial skin blisters. Exfoliation of the skin is caused by exfoliative toxins, types A and B (ETA and ETB). These toxins are excreted by the staphylococci and lead to the cleavage (of segmentation) of desmoglein complex 1. Disintegration of the desmosomes anchoring the strata granulosa [3] causes exfoliation. Not all phage types of *Staphylococcus* produce toxins [4]. The *Staphylococcus* which initially causes the disease is often not found in the biopsies or cultures, but usually originates from the nasopharynx. The condition causes severe morbidity and mortality if not treated promptly and correctly. In adults, mortality rates as high as 60% have been reported; in children mortality is generally lower, 4% [5].

Complications of SSSS include pneumonia, dehydration, and sepsis, albeit rare. Dehydration can lead to electrolyte imbalances; therefore, fluid management and laboratory monitoring are key. Some clinics include lactulose as a treatment as toxins are excreted gastrointestinally, especially in babies who lack mature kidneys. Prompt diagnosis and treatment halt further exfoliation and prevent morbidity and mortality. *Staphylococcus aureus* is usually susceptive to flucloxacillin. With the rise of multi-resistant strains such as methicillin-resistant *Staphylococcus aureus* (MRSA), the initial antibiotic regimen is due to change. In this case we also included clindamycin because this inhibits the toxins which cause the exfoliation.

SSSS can mimic other exfoliating diseases such as TEN and SJS. Differentiation is mainly done by assessing mucosal involvement. Furthermore, a lack of dermal inflammation indicates SSSS as TEN and SJS cause full thickness exfoliation. The diagnosis is often made by exclusion if there is exfoliation in the absence of signs of infiltration [6, 7]. Specific techniques such as polymerase chain reaction (PCR) tests to identify specific toxins are not readily available in most clinical settings [8].

Further studies should be conducted to assess the potential benefit of clindamycin. In addition, studies should be conducted into staphylococcal subtypes, as only a small portion excrete toxins. Knowledge of toxin excretion could have clinical implications. Lack of evidence and possible worsening or initiation of dehydration and electrolyte imbalances were the reasons for us not to include lactulose. Further research is warranted to assess a possible positive effect of standard lactulose treatment, especially in children. This case report highlights the lack of evidence which is needed to formulate evidence-based international guidelines.

## Conclusions

This case report highlights SSSS and its challenges in diagnosis and treatment. Therapeutic options and differential diagnosis are discussed. This case report highlights the successful therapy in our patient. Some treatment options are widely used without thorough research bases or guidelines. Further research is warranted and this case report aims to further research in exfoliating skin disorders.
